# Green and Yellow Pigments

**Published:** 1869-11

**Authors:** 


### GREEN AND YELLOW PIGMENTS.

Hitherto the chief source of the best and most brilliant green pigments has been some salt of copper, and especially a compound of that metal with arsenic. This has been largely used for paper staining, the manufacture of artificial leaves and flowers, and in dying materials for female dress. In a specimen of green tarlatan, thus dyed, M. Bobierre found no less than 391 grammes of arsenite of copper, or more than three-quarters of a pound, a considerable portion of which would, on the least shaking of the dress, fly into the air to the certain danger of surrounding persons. Among many attempts to provide non-poisonous pigments of equal brilliancy, perhaps the most successful are yellow zinc and green zinc, invented by Mr. Pettit. A most eminent chemical authority declares them as non-poisonous, not discolored by the effects of burning gas; not affected by hydrosulphuric acid, and unaltered by the action of light. They are capable of use in chromo-lithography, paper staining, as pigments, and for various other purposes.

